# Age at onset of Huntington disease is not modulated by the R72P variation in TP53 and the R196K variation in the gene coding for the human caspase activated DNase (hCAD)

**DOI:** 10.1186/1471-2350-6-35

**Published:** 2005-10-03

**Authors:** Larissa Arning, Peter H Kraus, Carsten Saft, Jürgen Andrich, Jörg T Epplen

**Affiliations:** 1 Department of Human Genetics, Ruhr-University, 44780 Bochum, Germany; 2 Department of Neurology, St. Josef-Hospital, Ruhr-University, 44791 Bochum, Germany

## Abstract

**Background:**

*TP53 *is an attractive candidate for modifying age of onset (AO) in Huntington disease (HD): The amino-terminus of the mutated huntingtin (htt) exon 1 translation product has functional properties which may affect critically the TP53 pathway in HD neurons. The pathogenic domain of mutant htt interacts with nuclear transcription factors, and it potentially modulates TP53-induced transcriptional events. A single nucleotide polymorphism (SNP) resulting in the R72P exchange in TP53 protein might modulate the variation in AO. In addition, also the R196K replacement in human caspase activated DNase (*hCAD*) may theoretically affect the AO.

**Methods:**

We have genotyped the polymorphisms R72P and R196K in a well established cohort of 167 unrelated HD patients.

**Results:**

The expanded CAG repeat explained 30.8% of the variance in AO. Adding the genotypes of the SNPs investigated did not affect the variance of the AO variance explained.

**Conclusion:**

In this replication study, no association was found explaining a significant amount of the variability in AO of HD thus contradicting a recent report.

## Background

Huntington disease (HD) is an autosomal dominantly transmitted, neurodegenerative disorder characterized by motor abnormalities, cognitive dysfunction and psychiatric symptoms [1]. HD is caused by an expansion of a polyglutamine tract in the amino-terminal portion of the protein huntingtin (htt), which apparently acquires a deleterious gain of function [2]. The length of the polyglutamine tract is the most important factor in determining AO of HD, although substantial variability remains after controlling for repeat length, particularly in cases where CAG repeat numbers range in the high 30 s or low 40 s [3]. Therefore, defining independent AO modifying factors is of great importance, since they may provide further clues pertaining to the pathology arising from the expanded repeats. In mammals, *TP53 *is the most important tumor suppressor gene. A variety of intracellular stress signals activate TP53 to induce either transient cell cycle arrest with stimulation of repair activities, senescence or apoptosis [4]. It has been proposed that the amino-terminal portion of mutant htt encoded by exon 1 exertsfunctional properties with significant consequences for the TP53 pathway in HD neurons. The pathogenic domain of mutant htt interacts with critical transcription factors and potentially modulates TP53-induced transcriptional events [5]. It has been shown that the two variations of the common codon 72 polymorphism (*Arg */*Pro*) in the *TP53 *gene differ with respect to the ability to suppress cellular transformation [6] and induce apoptosis [7]. Therefore, the R72P polymorphism represents a good candidate for modulating the AO of HD. Recently this polymorphism was described to explain a significant part of the variance in the AO in HD [8]. In the same study also the SNP R196K was investigated in the human caspase activated DNase protein (*hCAD*), since DNase is responsible for DNA degradation during apoptosis [9]. Variations in the latter SNP were likewise found to exert an additionally significant impact on the variation of AO. We reinvestigated here whether *TP53 *and *hCAD *variations modulate the AO of HD in our established cohort.

## Methods

Our study population consists of 167 unrelated patients with the clinical diagnosis of HD [10], recruited from the Huntington Center (HZ) NRW, Bochum (Germany). Clinical assessment and determination of the motor AO was performed exclusively by two experienced neurologists/psychiatrists of the HZ NRW. Controls (healthy blood donors from the German population of Essen) were also genotyped for comparison. Informed consent was obtained from all patients and controls. We purposely selected a cohort with limited extent of CAG expansions (41–45), since within this defined range the expansions show linear correlation with AO. Within this range ~75% of all elongated CAG alleles are comprised, and they are distributed normally. CAG repeat sizes were determined after PCR amplification of genomic DNA from peripheral white blood cells. CAG repeats were amplified by established methods [11]. The polymorphism R72P in the *TP53 *gene was demonstrated after PCR amplification using the following primers: 5'-GAGGACCTGGTCCTCTGACT-3' and 5'-GTAGGTTTTCTGGGAAGGGA-3'. PCR was carried out in a final volume of 10 μl with 50 ng of DNA, 200 μM dNTP and 1 U Taq Polymerase. Thermal cycling was performed with an initial denaturing step for 5 minutes at 94°C.; 35 cycles of denaturation at 94°C for 1 min, annealing at 55°C for 1 min and extension at 72°C for 1 min; and a final extension at 72°C for 10 min. PCR products were digested with the restriction enzyme Bsh1236I I at 37°C overnight and visualised on 2% agarose gels stained with ethidium bromide. This digestion yielded altogether 3 bands of different sizes: a 254 bp fragment (restriction site absent) corresponding to the C allele as well as a set of 160 and 94 bp fragments corresponding to the G allele (restriction site present). The polymorphism R196K in the *hCAD *gene was demonstrated using the following primers: 5'-CCTCTGACCACAGGACTGG-3' and 5'-TCTGTCGAAGTACGTGCCAT-3' under the same conditions mentioned above. PCR products were digested with the restriction enzyme Alu I at 37°C overnight. Digested DNA was electrophoresed on 3% agarose gels. The restriction fragments of the G allele were 129, 99 and 13 bp in length. The A allele harbors an additional restriction site so that the 99 bp fragment is digested into 73 and 26 bp. The variability in AO attributable to the CAG repeat number was calculated by linear regression. For the regression analysis, we used the AO as the dependent variable, the respective genotypes as independent variables.

The CAG repeat number was considered as numerical variables, the other putative modifying genotypes were considered as nominal variables. SPSS Ver.11.0 for Windows (SPSS Inc.) was used for all statistical analyses.

## Results and discussion

As detailed in an earlier study concerning our clinically thoroughly characterised HD cohort, the expanded CAG repeat explained 30.8% of the variance in AO [10]. Adding the genotypes of the SNPs investigated here did not affect the variance of the AO variance explained (Table 1). Chattopadhyay et al. further conducted a case-control study for these SNPs and found genotype GG in the *TP53 *gene to be a significant risk factor for HD. In order to retest also this assumption, we performed a case-control study as well. As expected, there was no difference between the case and control groups. All frequencies observed were in Hardy-Weinberg equilibrium and correspond to those reported for the general Caucasian population [12].

## Conclusion

Our study failed to replicate the association between the genotypes at the R72P polymorphism in *TP53 *and R196K polymorphism in *hCAD *genes with the AO of HD. Since we employed a larger cohort of exceptionally well-characterized HD patients the initial association may have been due to chance or to bias as introduced by population stratification. It is, however, possible that a true association exists in Indians. The effect size may also be quite small, requiring an even larger sample size than ours for Caucasians.

## Competing interests

The author(s) declare that they have no competing interests.

## Authors' contributions

LA initiated the study, carried out the molecular genetic studies and drafted the manuscript. PHK participated in the data analysis. JA and CS had ascertained the clinical status of the patients, and JTE participated in the study design, the coordination and finalized the analyses as well as the paper.

## Pre-publication history

The pre-publication history for this paper can be accessed here:



## Figures and Tables

**Table 1:** Genotype frequencies and linear regression analysis of the SNPs in *TP53 *and *hCAD *genes

**Gene**	**Genotypes**	**Number (%)**		**Gene (polymorphism)**	**R**^2^	Δ**R**^2^	**Unexplained variance (%)**	**p value**
							
		**HD patients**	**Controls**					
TP53	GG	89 (53)	86 (51)	HD CAG	.308	-		
	CG	69 (41)	69 (41)					
	CC	9 (5)	13 (8)	HD CAG +				
				*TP53 *(R72P)	.314	.006	0.9	.130
hCAD	GG	141 (84)						
	AG	23 (14)		HD CAG +				
	AA	3 (2)		*hCAD *(R196K)	.305	.003	0.4	.590
